# lncRNAs involved in the Shade Avoidance Syndrome (SAS) in *Arabidopsis thaliana*

**DOI:** 10.1186/s12864-024-10718-z

**Published:** 2024-08-26

**Authors:** Irving Jair García-López, Aarón I. Vélez-Ramírez, C. Stewart Gillmor, Selene L. Fernandez-Valverde

**Affiliations:** 1 Unidad de Genómica Avanzada, Cinvestav, Irapuato, 36824 Guanajuato México; 2 Laboratorio de Ciencias Agrogenómicas, Escuela Nacional de Estudios Superiores Unidad León, Universidad Nacional Autónoma de México, León, 37684 Guanajuato México; 3 Laboratorio Nacional PlanTECC, Escuela Nacional de Estudios Superiores Unidad León, Universidad Nacional Autónoma de México, León, Guanajuato, 37684 México; 4 School of Biotechnology and Biomolecular Sciences, The University of New South Wales, Sydney, NSW 2052 Australia; 5 UNSW RNA Institute, The University of New South Wales, Sydney, NSW 2052 Australia; 6 Evolution & Ecology Research Centre, The University of New South Wales, Sydney, NSW 2052 Australia

**Keywords:** Shade Avoidance Syndrome, Long non-coding RNA, WGCNA, *Arabidopsis thaliana*

## Abstract

**Background:**

Plant long non-coding RNAs (lncRNAs) have important regulatory roles in responses to various biotic and abiotic stresses, including light quality. However, no lncRNAs have been specifically linked to the Shade Avoidance Response (SAS).

**Results:**

To better understand the involvement of lncRNAs in shade avoidance, we examined RNA-seq libraries for lncRNAs with the potential to function in the neighbor proximity phenomenon in *Arabidopsis thaliana* (*A. thaliana*). Using transcriptomes generated from seedlings exposed to high and low red/far-red (R/FR) light conditions, we identified 13 lncRNA genes differentially expressed in cotyledons and 138 in hypocotyls. To infer possible functions for these lncRNAs, we used a ‘guilt-by-association’ approach to identify genes co-expressed with lncRNAs in a weighted gene co-expression network. Of 34 co-expression modules, 10 showed biological functions related to differential growth. We identified three potential lncRNAs co-regulated with genes related to SAS. T-DNA insertions in two of these lncRNAs were correlated with morphological differences in seedling responses to increased FR light, supporting our strategy for computational identification of lncRNAs involved in SAS.

**Conclusions:**

Using a computational approach, we identified multiple lncRNAs in Arabidopsis involved in SAS. T-DNA insertions caused altered phenotypes under low R/FR light, suggesting functional roles in shade avoidance. Further experiments are needed to determine the specific mechanisms of these lncRNAs in SAS.

**Supplementary Information:**

The online version contains supplementary material available at 10.1186/s12864-024-10718-z.

## Background

Neighbor sensing integrates light perception and plant-plant interactions into differential growth. It occurs when the plant in question receives light reflected from neighboring plants in the far-red wavelengths (700–800 nm) instead of direct photosynthetically active red and blue sunlight (400–700 nm) [1]. Shade-intolerant plants such as Arabidopsis have evolved a mechanism for phenotypic plasticity known as the Shade Avoidance Syndrome (SAS) [1, 2]. SAS is characterized by the elongation of hypocotyls and petioles, a reduction in leaf area, inhibition of branching, a decrease in chlorophyll content, and an acceleration of flowering. The perception of red and far-red light depends mostly on Phytochrome A (PHYA) and Phytochrome B (PHYB). Phytochromes function as sensors and are found in two photo-reversible forms: Pr (red light absorption) and Pfr (far-red light absorption) [3]. At high R/FR ratios, PHYB is found in its active form (Pfr) and enters the nucleus to interact with several Phytochrome Interacting Factor (PIF) transcription factors, which are degraded by the 16 S proteasome [4–6]. At low R/FR ratios, PHYB returns to the cytoplasm in its inactive form (Pr) allowing PIFs to accumulate in the nucleus to induce the expression of genes responsible for plant growth.

Non-coding RNAs include thousands of long non-coding RNAs (lncRNAs), RNA molecules greater than 200 nucleotides (nt) that have low or no protein-coding potential [7, 8]. According to their genomic context, lncRNA transcripts can be derived from intergenic, intronic or coding (sense and natural antisense) regions. Like mRNAs, they can be spliced, have a 5’ cap, and be polyadenylated. In contrast to their mRNA counterparts, lncRNAs have poor sequence conservation and are generally expressed at low levels in a tissue-specific manner [9–11]. In mammals, lncRNAs are involved in cell biology, differentiation, and metabolism [12]. In Arabidopsis, lncRNAs are expressed under biotic and abiotic stresses [13]; regulate flowering during vernalization [14–16]; function as miRNA decoys [17, 18]; and act in the immune response against pathogens [19]. lncRNAs have also been identified in important crops such as *Oryza sativa* [20–22] and *Zea mays* [23], under nitrogen deficiency in *Populus* [24], in response to cold in *Medicago truncatula* [25], and in tomato during the infection by *Phytophthora infestans* [26], highlighting their role in responses to different types of biotic and abiotic stress.

Some evidence exists that lncRNAs also act in photomorphogenesis: about 13,000 white light-responsive NATs lncRNAs were identified in cotyledon and hypocotyl tissues of Arabidopsis [27], and lncRNAs have been shown to be expressed in the late stage of SAS in *Dendrobium officinale* [28]. The most important example of lncRNA growth regulation by light is *HIDDEN TREASURE 1 (HID1)*, an intergenic lncRNA that represses the transcription factor *PIF3* under continuous red light, acting as a positive regulator of red light-mediated photomorphogenesis [29]. The *BLUE LIGHT INDUCED LONG NON-CODING RNA (BLIL1)* is induced by blue light and acts as a decoy for miRNA167, which inhibits hypocotyl growth [18]. Recently, *PHYA UTR ANTISENSE lncRNA (PUAR)* was shown to promote shade-dependent hypocotyl elongation by repressing *PHYA* [30].

In this work, we sought to identify lncRNAs expressed under shade conditions, indicating they might be involved in the SAS response. We identified differentially expressed lncRNAs in cotyledon and hypocotyl tissues of Arabidopsis seedlings subjected to high R/FR (control) and low R/FR (shade) light conditions [31]. We constructed weighted gene co-expression networks to infer a biological function for these differentially expressed lncRNAs by identifying co-expression modules with SAS-related functions, leading to a set of three lncRNAs likely involved in SAS. T-DNA insertions in two of these three lncRNA genes were correlated with altered R/FR responses in seedlings, suggesting that these two lncRNAs play a functional role in the SAS.

## Methods

### Filtering, assembly, and quantification of transcripts across all transcriptomes

We assembled and quantified all transcripts in early shade response transcriptomes from cotyledon and hypocotyl [31] following the methodology from our previous work [32].

### lncRNA identification and classification by genomic position

LncRNAs were discriminated from the coding genes using a Strict Method, removing the transcripts with a larger ORF, signal peptide, and known proteins, as described previously [32]. They were subsequently annotated according to our previous work, using their genomic position in relation to other genes [32]. To better contextualize our findings, we used previous studies targeting lncRNAs in Arabidopsis and, when possible, in the context of light experiments or phytochrome alterations. Specifically, lncRNAs with names in the format *AtRegRNA.00000* are from [32], those with names similar to *AT0TU000000* are from [27], those named *NAT-lncRNA_000* were annotated by [33], and those listed as *LNC_000* are from [30]. LncRNAs with names in the well-known *AT0G00000* format are part of the Araport11 annotation [34].

### Differential expression and functional annotation

Differential expression analysis was performed with the library DEseq2 [35] using the treatment: time interaction model. Four contrasts were generated with the above model (0 vs. 15 min, 15 vs. 45 min, 45 vs. 90 min, and 90 vs. 180 min). A gene was considered as differentially expressed if it had an FDR < 0.01. Functional enrichment was performed with topGO [36] using the annotation of org.At.tair.db [37] and differentially expressed results, considering the annotated terms with a p-value < 0.01 yielded with a classic Fisher’s test.

### Construction of co-expression networks with WGCNA

The guilt-by-association approach was used to infer the possible function of lncRNAs. The co-expression network was constructed with WGCNA by performing a Variance Stabilizing Transformation (vst) of the count reads [35]. The 𝛽 parameter was determined by the scale-free network law. To ensure that the average connectivity of the network is continuous, a value of 𝛽 = 12 was chosen, which is the lowest value for which the scale-free topology index curve remains stationary. The signed network was signed by creating a matrix by means of a biweight midcorrelation (bicor) with the option of unmerged modules. Expression profiles were represented by their principal component (module eigengene), which indicates the variation of expression levels in each module [38].

For co-expressed modules, GO category enrichment analysis was performed with topGO for biological processes considering significantly annotated terms with *padj* < 0.01 [36]. GO enrichments for each co-expressed module were generated both with all genes and with DEGs genes in each module. For KEGG annotation KEGGREST [39] was used (ath pathways list) using the log2 fold changes of DEGs, and a Wilcox test was applied for gene annotation for each pathway with a *p. value* < 0.01.

### Identification of PIF binding sites

The lncRNAs upstream regions (2000 bp) were taken to examine the absence/presence of PIF binding sites using the current version of ReMap UCSC US site public tracks (2022) from *A. thaliana* [40]. The plyranges package was used with the function join_overlap_intersect to intersect the lncRNA putative promoter regions with the PIF ReMap regions [41].

### Plant material and growth conditions

Arabidopsis seeds for evaluation with T-DNA mutants in or near lncRNAs (SALK_127009, SALK_140097, SAIL_69_F12C1) were obtained from Arabidopsis Biological Resource Center (ABRC). Homozygous seeds were sterilized with 70% ethanol (v/v) for 5 min, then seeds were washed with 100% ethanol. Seedlings were grown on MS medium with 1% sucrose (w/v), 6% phytagel (w/v) and stratified for 2 days at 4 °C. The plates were put in a vertical position in a growth chamber for 4 days at 22° C and 16 h light / 8 h dark photoperiods (full spectrum BESTVA Pro 1000 W LED lights at 200 µmol $\:m$^−2^$\:s$^−1^), and then transferred to High R/FR and Low R/FR conditions for 3 days. Two chambers were assembled with LED UPDAYDAY (UD-2500HPS) lamps; one lamp was used without modification, while the other one was fitted with 96 FR LEDs (10 mm SinkPAD LuxeonStar with LXML-PF01 LEDs LUMILEDS, 720 nm). Both treatments were maintained at a photosynthetically active photon flux density of 150 µmol $\:m$^−2^$\:s$^−1^ at 22° C ± 1° C. Lamp spectra were measured using a SILVER-Nova Super Range TEC spectrophotometer, and the R/FR ratio was calculated at 5.83 for High and 0.28 for Low conditions. The phytochrome photostationary state (PSS) values were calculated as 0.85 and 0.62 for High and Low R/FR, respectively, following the method described by [42]. The measurements of the hypocotyls and petioles were performed with ImageJ software [43].

### Primers for T-DNA genotyping

LBb1.3 (ATTTTGCCGATTTCGGAAC) and LB3 (TAGCATCTGAATTTCATAACCAATCTCGATACAC) for SALK and SAIL lines, respectively, were used to amplify the genomic flanking region.

**Table:** 

T-DNA Line	Left primer (LP)	Right primer (RP)
**SAIL_69_F12C1**	TGGTTAATCTGACCATCTTTAAACTG	TCATATATCGGCATCAGGCTC
**SALK_140097**	CATAAACTTTGCTACTGGCCG	TTAAATCGATCGCATCTTTGG
**SALK_127009**	GGGAAACACAAAATCACATGC	TTGAAAACGAAGATGAGCCAG

## Results

### Differential expression of lncRNAs in high R/FR vs. low R/FR

**Fig. 1:**
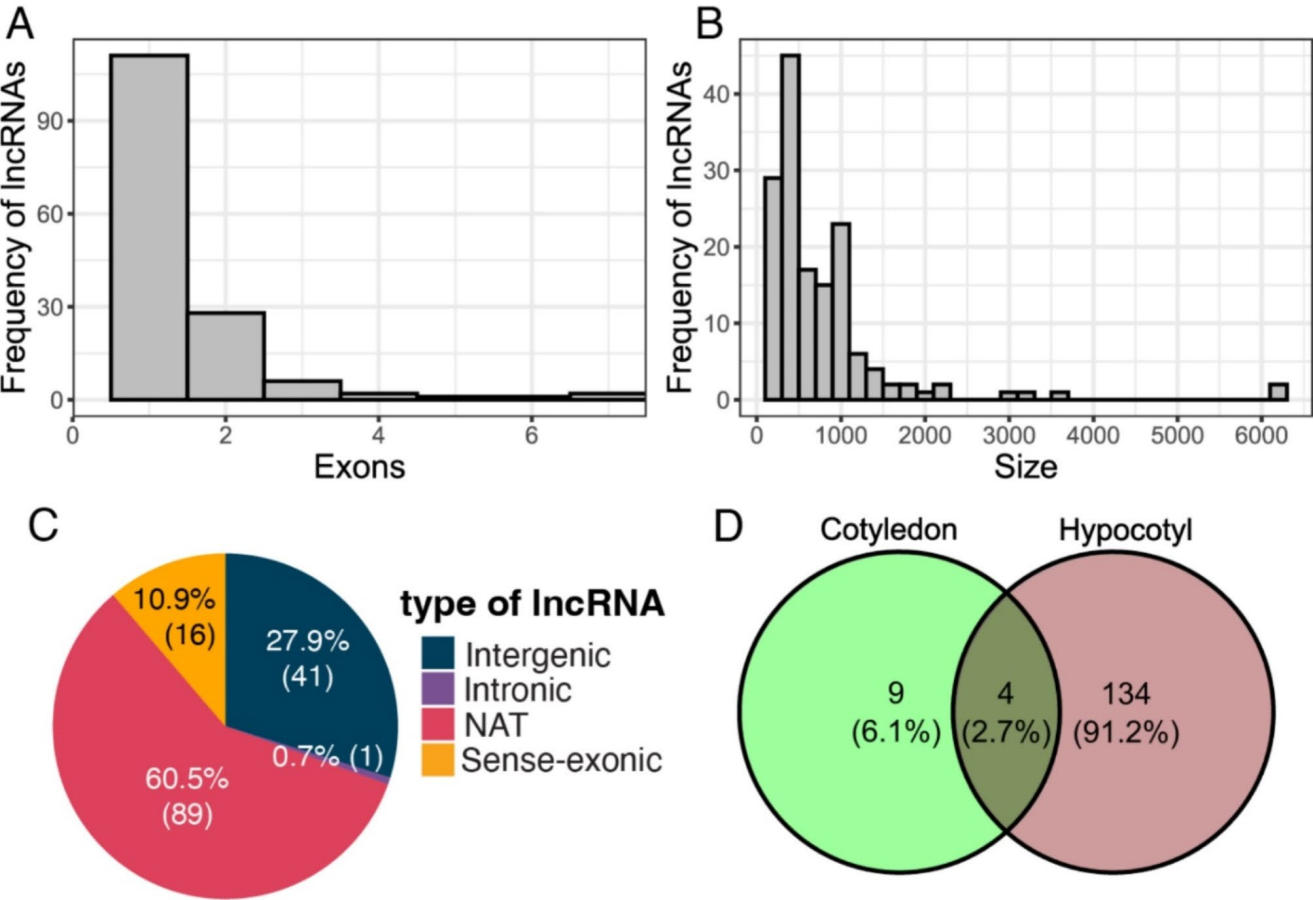
General characteristics of differentially expressed lncRNAs. Distribution of **a**) exon number, **b**) length in bp, **c**) classification by genomic context, and **d**) Venn diagram of DE lncRNAs in both tissues

We recently annotated 6,764 lncRNAs in Arabidopsis [32]. Using this reference set of lncRNAs along with the Araport11 annotation for coding genes, we performed a differential expression analysis of RNA-seq libraries of cotyledon and hypocotyl tissues of Arabidopsis seedlings subjected to high R/FR (control) and low R/FR (shade) conditions at four exposure times (15, 45, 90, 180 min) [31]. As measured by principal component analysis, in PC1 in the cotyledon, the main variance is given by the time rather than the treatment (66%), and in the hypocotyl, the variance observed (75%) between libraries was explained by the applied treatment in the last timepoints (Fig. S1). We performed contrasts to determine differentially expressed coding and non-coding genes between timepoints, focusing on the interaction of treatment and time in each tissue. Among all the timepoints, 1,056 differentially expressed coding genes were found in cotyledons, while 7,467 differentially expressed coding genes were found in hypocotyls (Table S1). The majority of the 147 differentially expressed lncRNAs consisted of a single exon (Fig. 1a) and had transcripts of less than 1000 bp in length (Fig. 1b). Of these, 89 were natural antisense transcripts (NATs), 41 were intergenic lncRNAs, 16 were sense-exonic transcripts, and one was an intronic lncRNA (Fig. 1c). In addition, 134 lncRNAs were found exclusively in the hypocotyl, 4 were found in both hypocotyl and cotyledon, and 9 were exclusively in the cotyledon (Fig. 1d).

**Fig. 2:**
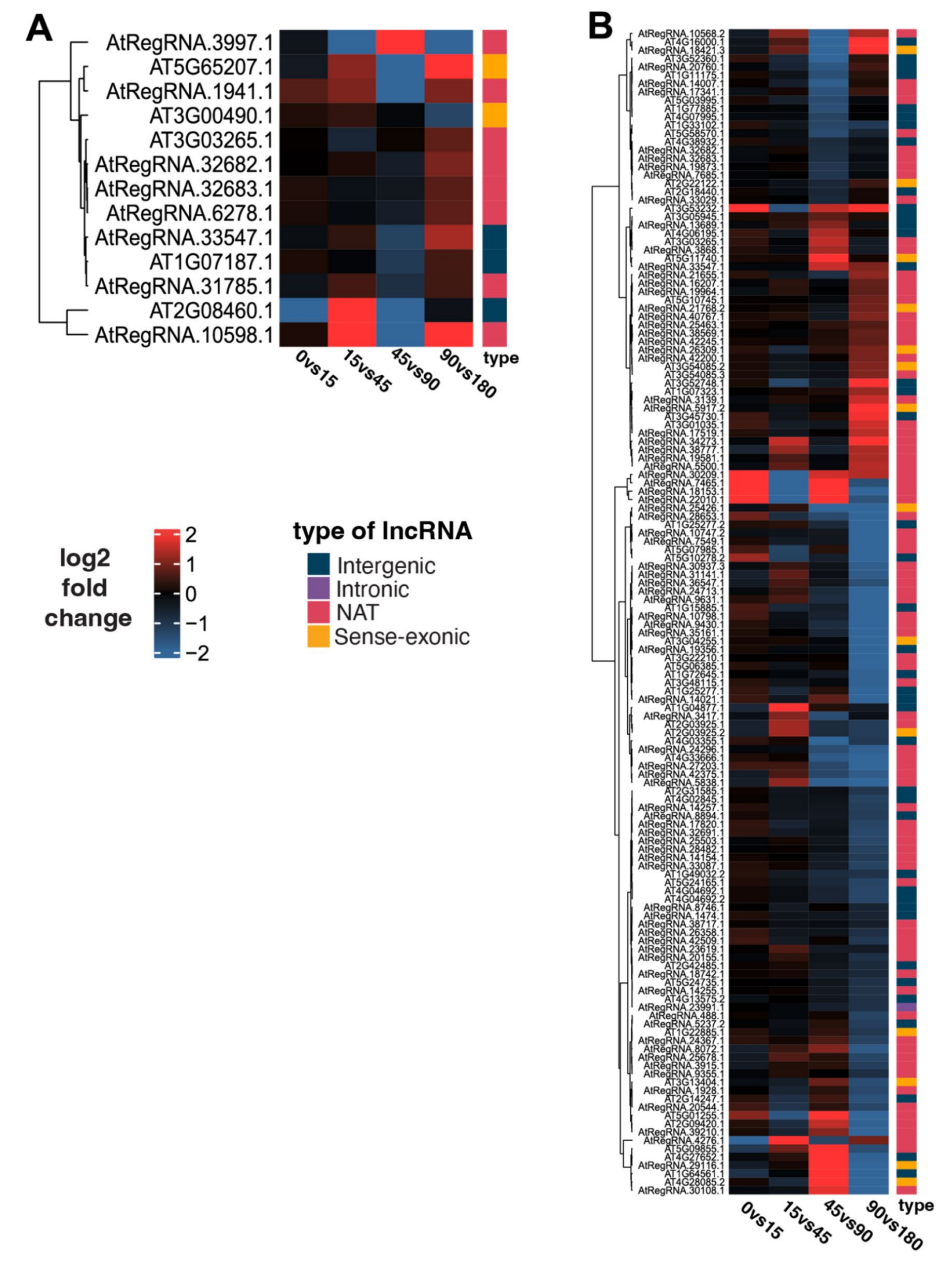
Differentially expressed lncRNAs by class. Separated heatmaps in **a**) cotyledon and **b**) hypocotyl during high and low R/FR light treatments. Each column represents a contrast of minutes vs. treatment. The bar annotation to the right represents each class of lncRNA. Differential genes using Wald-test *padj* < 0.01

Figure 2 shows all lncRNAs that were differentially expressed for at least one timepoint, and Table S2 specifies at which timepoint the lncRNA showed statistically significant differential expression. No lncRNAs were identified as differentially expressed between 0 and 15 min. Comparing 15 and 45-minute samples, one intergenic lncRNA and one NAT were induced in cotyledons, while in hypocotyl, one intergenic lncRNA and 2 NATs were induced. In contrast, one intergenic lncRNA and 3 NATs were repressed. At 45 vs. 90 min, one NAT was induced in cotyledon, and 3 NATs and 2 intergenic lncRNAs were repressed. Meanwhile, in hypocotyl, 14 lncRNAs were induced (6 NATs, 6 intergenics and 2 sense-exonic) while 13 intergenic lncRNAs, 19 NATs and 2 sense-exonic lncRNAs were repressed. At 90 vs. 180 min, one intergenic, one sense-exonic and 4 NAT lncRNAs were induced in cotyledons, and just one sense-exonic lncRNA was repressed; in hypocotyl, 49 NATs and 21 intergenic lncRNAs were repressed (Table S2).

Looking at all gene expression in cotyledons in shade vs. normal conditions (Table S1), 38 coding genes were induced between 0 and 15 min. In comparison, 286 genes were differentially expressed from 15 to 45 min, including 2 induced lncRNAs. In the third contrast (45 vs. 90 min), 386 genes were differentially expressed, including one induced and 5 repressed lncRNAs. From 90 to 180 min, 635 genes were differentially expressed, including 6 induced and one repressed lncRNA. In hypocotyl tissue, 24 coding genes were differentially expressed from 0 to 15 min. From 15 to 45 min, 254 genes were differentially expressed, including 3 induced and 4 repressed lncRNAs. From 45 to 90 min, the number of differentially expressed genes increased exponentially to 2,862, of which 14 were induced and 34 were repressed lncRNAs. In the 90 to 180-minute contrast, 6,059 genes were differentially expressed, including 77 repressed and 28 induced lncRNAs (Fig. 2). Of the differentially expressed coding genes, 315 were found only in cotyledon, while 9,106 were present in hypocotyl (Fig. 2 and Tables S1, S2).

In summary, by comparing hypocotyl and cotyledon tissue in seedlings shifted from a high R/FR to a low R/FR ratio, we identified 13 differentially expressed lncRNAs in cotyledons and 138 differentially expressed lncRNAs in hypocotyls. Differential expression of these lncRNAs in control vs. ‘shade’ conditions suggests they might be involved in the SAS response. Most of these are lncRNAs newly annotated by Corona-Gomez et al. [32], including 8 in cotyledon tissue and 84 in hypocotyl (Figs. 1d and 2).

### Gene ontology of differentially expressed genes

**Fig. 3:**
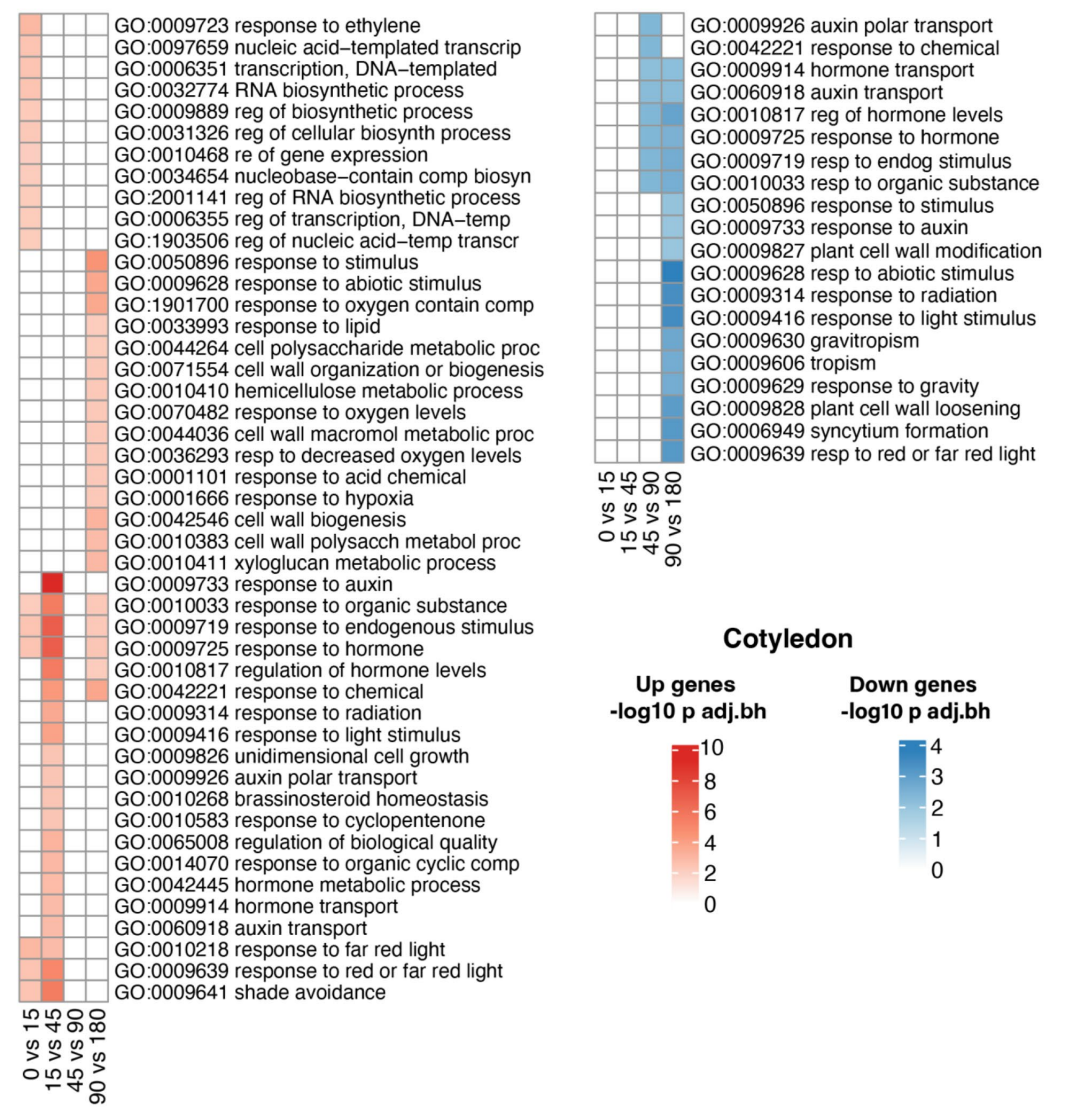
Functional enrichment of differentially expressed genes in cotyledons during shade treatment. GO terms of biological processes, -log10 of the p-value < 0.01 (Classic Fisher). **a**) induced and **b**) repressed gene terms

For genes differentially expressed between high and low R/FR conditions, we performed a functional enrichment for the ontology of biological processes in cotyledons and hypocotyl. In cotyledons (Fig. 3), overrepresented GO categories at the 0 vs. 15-minute contrast were related to light perception and transcription, including ‘response to red light’, ‘response to red or far-red light’, ‘shade avoidance’, ‘regulation of transcription’ and ‘regulation of gene expression’. At the 15 vs. 45-minute contrast, overrepresented terms were related to cell growth and relaxation of the cell wall, such as ‘auxin transport’, ‘hormone transport’, ‘regulation of hormone levels’ and ‘unidimensional cell growth’. No overrepresented categories were seen in the 45 vs. 90-minute contrast, while at the 90 vs. 180-minute contrast, overrepresented GO categories were mostly related to cell wall metabolism, such as ‘cell wall organization or biogenesis’, ‘cell wall biogenesis’, ‘hemicellulose metabolic process’ and ‘xyloglucan metabolic process’ (Fig. 3a). Thus, the first genes induced in cotyledons by low R/FR light are related to light perception and transcription, the next genes induced are related to cell growth, and later induced genes are related to cell wall remodeling. These differentially expressed genes are consistent with the perception of differential light conditions and subsequent transcriptional and biochemical responses. Genes repressed in cotyledons consisted primarily of genes upregulated at earlier stages, for example, ‘response to hormone’, ‘auxin transport’, ‘hormone transport’, and ‘response to light stimulus’. This suggests that the transcriptional responses to changes in R/FR ratios fluctuate quickly (Fig. 3b).

**Fig. 4:**
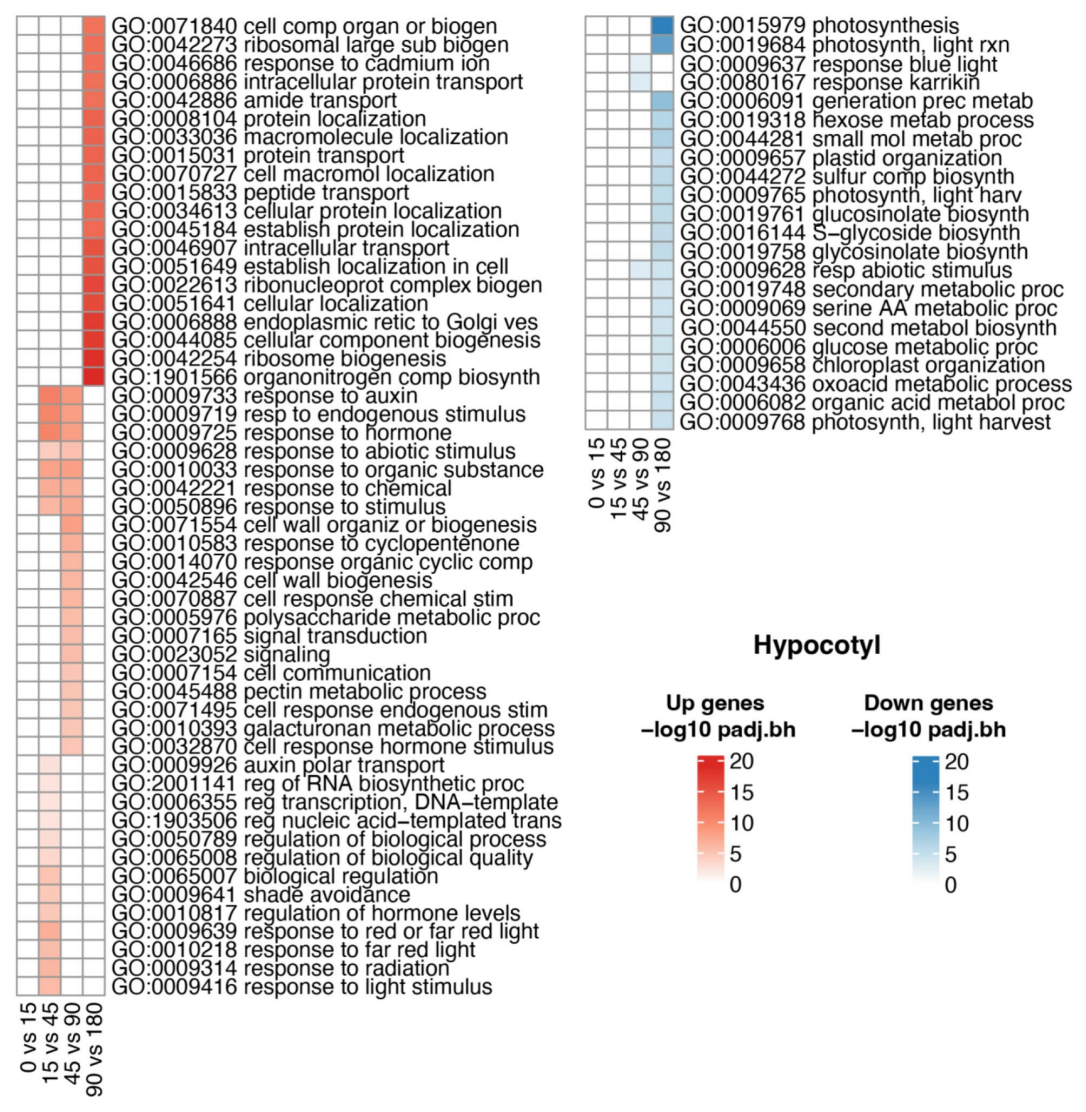
Functional enrichment of differentially expressed genes in hypocotyl during shade treatment. GO terms of biological processes, -log10 of the p-value < 0.01 (Classic Fisher). **a**) Induced and **b**) repressed gene terms

In hypocotyl (Fig. 4), GO categories for induced genes were similar to those in cotyledons: “Response to hormone”, “endogenous stimulus”, “organic substance”, ‘response to auxin’, and ‘chemical and abiotic stimulus’. Processes such as ‘red and far-red light signaling pathway’, ‘far-red light response’ and ‘shade avoidance’ were significantly induced in the first few minutes of exposure. At intermediate times, processes such as ‘cell wall biogenesis’ and ‘metabolic process of cell wall components’ were also overrepresented (Fig. 4a). In the case of the genes repressed in hypocotyl, processes deregulated in the last two contrasts related to ‘response to abiotic stimulus’ and ‘photosynthesis’ (Fig. 4b).

### Functionally contextualizing lncRNAs using gene co-expression networks

**Fig. 5:**
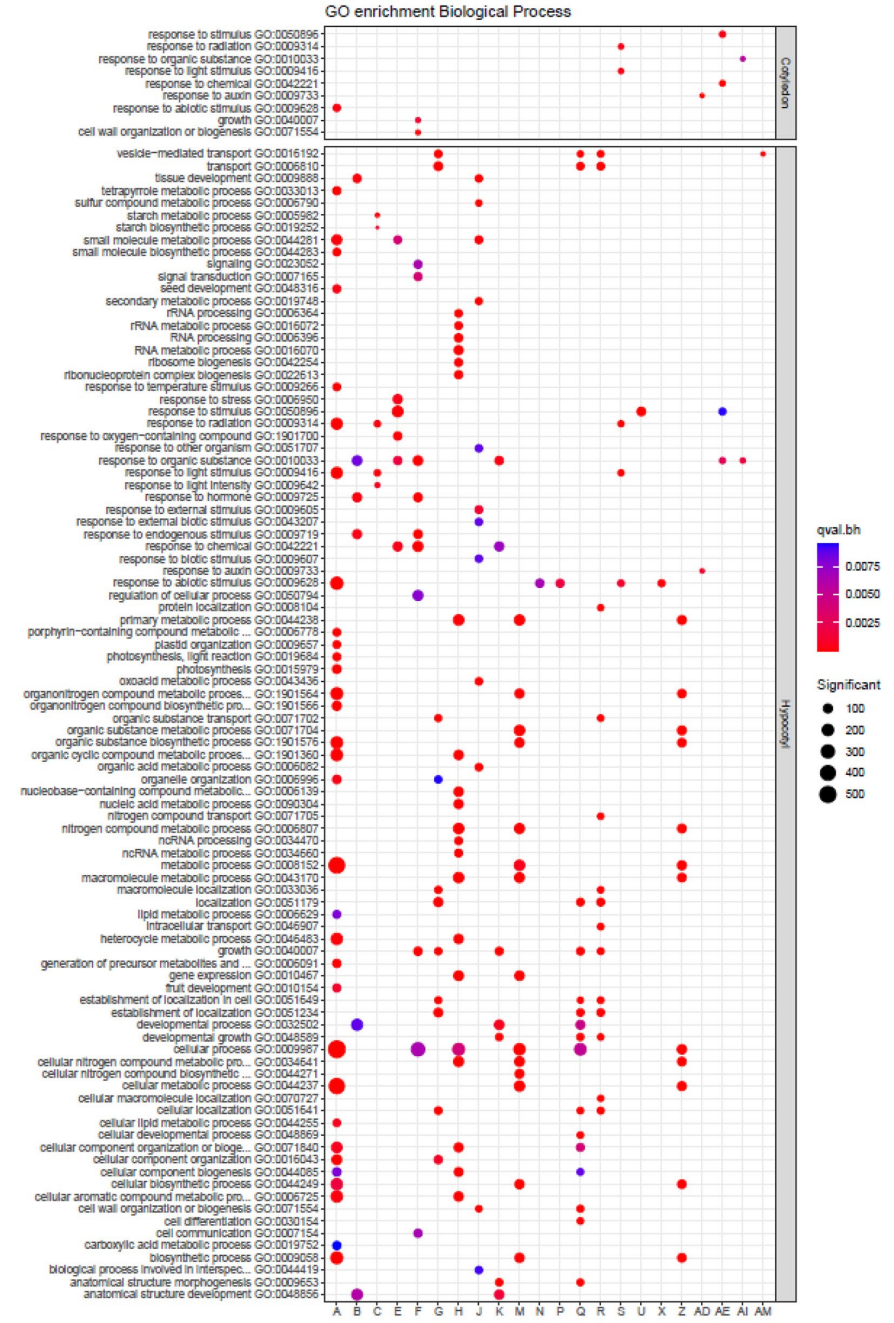
GO enrichment for gene co-expression modules. Coexpressed modules of DEGs with biological process enrichment in one or both tissues. The network was built using all the genes. Only GO enrichments for the DEGs genes < 0.01 p.adj are shown. All modules are shown in Fig. S1

To identify the biological processes in which the identified lncRNAs might be involved, we constructed a weighted gene co-expression network resulting in 39 modules of highly co-expressed genes (Supplemental Fig. 2). Module A (turquoise) was the largest module with 3,325 co-expressed genes, while the smallest was Module AM (orangered4) with 22 co-expressed genes. For each module, we used functional and KEGG enrichments to assign a set of putative biological functions to the coding and lncRNA genes in the module. Twenty-nine modules presented specific functional enrichments; Fig. 5 shows the 22 modules containing both DEGs and lncRNAs (not all modules in Fig. 5 had DE lncRNAs, but all modules contained lncRNAs). Modules with biological processes related to the neighbor-proximity phenomenon included modules A (GO terms: ‘response to radiation’, ‘response to light stimulus’, ‘response to abiotic stimulus’, ‘cellular component organization’), B (GO terms: response to ‘hormone‘, ‘developmental process‘, ‘anatomical structure development‘), F (GO terms: ‘cell wall organization or biogenesis’, ‘response to hormone’, ‘growth’), G (GO term: ‘growth’), J (GO term: ‘cell wall organization or biogenesis’), K (GO terms: ‘growth’, ‘developmental process’, developmental growth’, ‘anatomical structure morphogenesis’), Q (GO terms: ‘anatomical structure morphogenesis’, ‘Cell differentiation’, ‘cell wall organization’, ‘developmental growth’, ‘developmental process’, ‘growth’), R (GO terms: ‘developmental growth’, ‘growth’), S (GO terms: ‘response to light stimulus’) and AD (GO term: ‘response to auxin’).

**Fig. 6:**
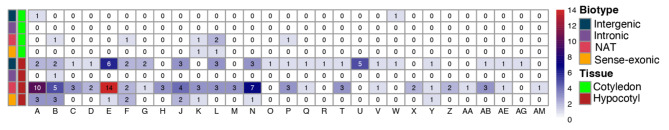
Modules that contain DE lncRNAs in both cotyledon and hypocotyl. Differentially expressed lncRNAs for each module are shown, including biotypes and tissues. Primary data is found in Table S4

In cotyledons, differentially expressed lncRNAs were found to be co-expressed with coding genes from 7 modules (Fig. 6). However, not all lncRNAs in these modules were differentially expressed. For example, in cotyledon, module K was enriched in processes such as ‘growth’, ‘developmental process’, ’developmental growth’, and ‘anatomical structure morphogenesis’, but we found only one differentially expressed NAT and one sense-exonic lncRNA out of a total of 55 lncRNAs in module K (Table S3, S4). Module L harbored the most DE lncRNAs (2 NAT and one sense-exonic). Other modules with differentially expressed lncRNAs included A, B, F, P and W with only one differentially expressed lncRNA found in each (Fig. 6).

In hypocotyl tissue, we found differentially expressed lncRNAs distributed in 29 modules. We found 6 DE lncRNAs in module F (2 NAT, 2 sense exonic and 2 intergenic), 6 DE lncRNAs in module L (3 NAT and 3 intergenic), and 11 DE lncRNAs in module B (with GO Terms involved in cell wall processes). Module A had 15 DE lncRNAs related to ‘metabolic and cellular process’ and ‘photosynthesis’, while only 1 DE lncRNA was present in cotyledon. Ten modules (I, S, AC, AD, AF, AH, AI, AJ, AK, AL) did not have DE lncRNAs in at least one tissue, and five modules did not have any lncRNA (AF, AH, AJ, AK, AL) (Fig. 6 and Table S3).

PHYTOCHROME INTERACTING FACTOR (PIF) transcription factors are among the most important transcription factors for light regulation of growth and morphogenesis in plants [4–6]. We analyzed upstream putative regulatory regions (2,000 bp upstream of the lncRNA) of the DE lncRNAs in shade conditions to look for PIF binding sites (PIF1, PIF3, PIF4, PIF4 in etiolated plant experiments, PIF5 and PIF7). We found 9 lncRNAs in cotyledon and 109 in hypocotyl with at least one PIF binding site in their upstream region (Fig. S3). The existence of PIF binding sites in so many of the shade-responsive lncRNAs identified in our study provides evidence that these lncRNAs may indeed function in the regulation of seedling responses to light conditions.

Further evidence for the efficacy of our approach to finding shade-regulated lncRNAs is the fact that we found a few lncRNAs that have been previously identified as changing expression in response to light conditions. This includes an antisense lncRNA (NAT) *AtRegRNA.19,581* (Module F), which is upregulated in hypocotyl between 90 and 180 min and a NAT (*AT3TU012440*) was found nearby previously [27]. *AtRegRNA.19,581* has *PIF3* and *PIF7* motifs in its upstream region and overlaps with *CLATHRIN HEAVY CHAIN 1 (CHC1)* (*AT3G11130*) in module R and *LOB DOMAIN-CONTAINING PROTEIN 21 (LBD21) (AT3G11090*) in module P. *LBD21* is downregulated concurrently with the NAT (90 vs. 180 min contrast) and is enriched in response to abiotic stimulus. *AtRegRNA.19,581* was co-expressed with *EXPANSIN 11 (EXP11)*,* ARABINOGALACTAN PROTEIN 2 (AGP2)*,* REVERSIBLY GLYCOSYLATED POLYPEPTIDE 1 (RGP1)*, *CELLULOSE-SYNTHASE LIKE C5 (CSLC5)*, *GALACTURONOSYLTRANSFERASE 6 (GAUT6)*,* GAUT9*, all genes encoding proteins involved in cell wall remodeling and plant morphogenesis. The co-expression of *AtRegRNA.19,581* and these cell wall remodeling genes is consistent with a role for this lncRNA in differential growth in response to shade conditions.

### Description of modules with differentially expressed lncRNAs

Module A contains 257 lncRNAs and is the module most enriched in genes related to response to light. *AtRegRNA.14021* (- strand) is a new intergenic lncRNA downregulated in hypocotyl at the final timepoint of shade treatment (90–180 min), whose promoter is bound by PIF1 and PIF4. Interestingly, *1-AMINOCYCLOPROPANE-1-CARBOXYLATE SYNTHASE 4 (ACS4)*, a gene involved in ethylene biosynthesis that is induced by auxin and part of module S, is located upstream of *AtRegRNA.14,021*. Like *AtRegRNA.14021*, *ACS4* is downregulated at the 90–180 min contrast. The previously identified NAT-lncRNA_1535 overlaps with *AtRegRNA.14021* [33].

In module B, we identified NAT *AT2G09420* as upregulated between 45 and 90 min and downregulated between 90 and 180 min in hypocotyl. This lncRNA was co-expressed with *XYLOGLUCAN ENDOTRANSGLUCOSYLASE/HYDROLASE 4 (XTH4)* and overlaps the 3´ end of the transcription factor *BETA HELIX LOOP HELIX 129 (BHLH129)* (*AT2G43140*), which is downregulated at final timepoints, suggesting that NAT *AT2G09420* could act as a repressor of its *cis* gene. The gene downstream of NAT *AT2G09420*, *ARABIDOPSIS RAB 4 (ARA4)* (involved in vesicle transport and part of module Q), was upregulated at the same timepoint as *BHLH129* was downregulated. A NAT that partially overlaps NAT *AT2G09420* was previously reported (*NAT-lncRNA_1505*), while NAT *AT2TU072100* was previously identified in the body of *BHLH129* [25, 27]. *AT2G09420* was co-expressed with upregulated genes in the same contrast as *INDOLE-3-ACETIC ACID INDUCIBLE 9* (IAA9), *EXP4*, *HERCULES RECEPTOR KINASE 2* (*HERK2*) and *DWARF IN LIGHT 1* (*DFL1*).

Module B also contained *AtRegRNA.1474.1*, a NAT lncRNA that overlaps *SHORT OPEN READING FRAME 1 (SORF1)*, a translated small open reading frame of unknown function. *AtRegRNA.1474.1* has PIF3 and PIF4 DNA binding motifs in its upstream region (Supplemental Fig. 3) and was downregulated in hypocotyls at the 90–180 min contrast. *AtRegRNA.1474.1* was identified previously (*AT1TU012380*) [27]. Finally, we identified *AtRegRNA.8746* to be differentially expressed in hypocotyls at the 90 vs. 180 min contrast, and to have both upstream and downstream PIF3 DNA binding motifs. *AtRegRNA.8746* was originally annotated as a lincRNA, but it overlaps with the sense lncRNA *AT1G08877* that has *MIR157A* in its first exon. *AtRegRNA.8746* was co-expressed with downregulated genes in the same contrast as *IAA13* (Auxin induced gene) and *INDOLE-3-ACETIC ACID INDUCIBLE 27* (*IAA27*), *HERK2*, *LIKE AUXIN RESISTANT* (*LAX2*, *LAX3*), *EXP4*, and *GRAVITROPIC IN THE LIGHT* (*GIL*).

Genes in module E gradually and then drastically decreased their expression in the hypocotyl under low R/FR across the time course, and all lncRNAs co-expressed in this module were downregulated in the final point of treatment. We identified NAT lncRNA *AtRegRNA.31141* as downregulated in hypocotyl at 90 vs. 180 min. *AtRegRNA.31141* has upstream binding sites for PIF3, PIF4, PIF5 and PIF7, and overlaps the 3’ end of its *cis* gene *MEMBRANE OF ER BODY 1 (MEB1)* (*AT4G27860*), a vacuolar iron transporter family protein. Other NATs also overlap the *MEB1* gene: *AT4G27852* overlaps the 5’ end, and NATs *AT4TU056710* and *NAT-lncRNA_3651* overlap the gene body [27, 33]. *AtRegRNA.31,141* was co-expressed with genes such as *CSLA10*, *SAUR31*, *SAUR67*, *CXC750*, *AUXIN RESPONSE TRANSCRIPTION FACTOR 3 (ARF3)*, *XYLOGLUCAN ENDOTRANSGLUCOSYLASE/HYDROLASE 31 (XTH31)* and *XTH32*. Further, the NAT lncRNA LNC_5138 that overlaps *MEB1* increases its expression during shade treatment in *pif7* mutants [30].

Module F displays one of the most drastic increases in gene expression in hypocotyls in response to low R/FR conditions and is enriched in GO terms for ‘growth’, ‘cell morphogenesis’, ‘cell wall biogenesis’, ‘response to hormone’, ‘Golgi related’ and ‘developmental processes’ (Fig. 5, Fig. S4). Module F contains the NAT *AtRegRNA.1941*, a lncRNA downregulated in cotyledon at the middle contrast (45–90 min) (Table S4). *AtRegRNA.1941* overlaps the 3’ end of *SBH2* and has PIF3, PIF4, PIF5 and PIF7 DNA binding motifs upstream of its transcriptional start site. Genes involved in auxin responses such as *SMALL AUXIN UP RNA 19 (SAUR19)*,* SAUR26*,* SAUR27*,* SAUR29* are downregulated at the same timepoint as *AtRegRNA.1941*, suggesting that this lncRNA is involved in light-regulated growth.

Module F also contains the intergenic lncRNA *AT1G04877* (annotated by Araport11) that has a PIF3 binding site in its upstream region. In hypocotyl, *AT1G04877* was upregulated at the 15–45 min contrast along with genes such as *SAUR19*,* SAUR26*,* SAUR27*,* SAUR28*,* SAUR29*,* SAUR68*,* BASIC LEUCINE-ZIPPER 52 (BZIP52)*, *ROTUNDIFOLIA LIKE 13 (RTFL13)*, *AUXIN-REGULATED GENE INVOLVED IN ORGAN SIZE (ARGOS)*, *AUXIN SIGNALING F BOX PROTEIN 1 (AFB1)*, *INDOLE-3-ACETIC ACID INDUCIBLE 14 (IAA14)*, *GH3.5*, *ARABIDOPSIS THALIANA HOMEOBOX 3 (ATHB-3)*, *BETA HELIX LOOP HELIX 134 (BHLH134)*, *IAA4* and *FRAGILE FIBER 1 (FRA1)*. These genes are involved in GO terms such as ‘cell wall organization and biogenesis’, ‘developmental growth’, ‘developmental process’, ‘response to auxin’ and ‘response to hormone’. RTFL13 has recently been shown to negatively regulate the shade avoidance response by interacting with BRASSINOSTEROID SIGNALING KINASES [44]. Two genes adjacent to lncRNA *AT1G04877* are also regulated by R/FR light responses: the downstream ankyrin repeat family protein *AT1G11740* (part of module O, upregulated at 45 vs. 90 min) and the upstream gene *STARCH SYNTHASE 3 (SS3)* (part of module A, downregulated at 90 vs 180 min). We found the NAT lncRNA *AtRegRNA.4276* to be co-expressed with lncRNA *AT1G04877*. The upstream region of *AtRegRNA.4276* has DNA binding motifs for PIF3, PIF4, PIF5 and PIF7, and *AtRegRNA.4276* overlaps the 3’ end of the adjacent methyltransferase superfamily gene *AT1G29470*, which was upregulated at the 90 vs. 180-minute contrast. *SAUR68* is found 10 kb downstream of *AtRegRNA.4276* and was coexpressed with *AtRegRNA.4276*.

Finally, in module F, we identified the sense-exonic lncRNA *AtRegRNA.29,116*. *AtRegRNA.29,116* has an upstream PIF3 binding site and overlaps the glycine-rich protein gene *AT4G15150*, which is induced in hypocotyl at the 45 vs. 90 min contrast. *AtRegRNA.29,116* was coexpressed in the same contrast with genes like *BETA-XYLOSIDASE 2 (BXL2)*, *SAUR1*, *SAUR4*, *SAUR9*, *SAUR26*, *SAUR29*, *SAUR35*, *SAUR68*, *SAUR77*, *ARF11*, *EXPANSIN 11 (EXP11)*, *XTH17*, *XTH30*, *PIN FORMED 3 (PIN3)*, *CSLC4*, *CSLC5*, *CSLC8*, *ARGOS*, *IAA14*, *IAA4*, *GH3.1*, *GH3.5*, and *ARABINOGALACTAN PROTEIN 4 (AGP4)*. This lncRNA was previously annotated as NAT *AT4TU037310* [27].

Genes in module K increase substantially in hypocotyls in low R/FR, with a minor decrease in expression in cotyledons. This module includes genes such as the A2-type cyclin *CYCA2;3*; the microtubule organizer *​​RHO-RELATED PROTEIN FROM PLANTS 2 (ROP2)*; cell wall remodelers *PECTIN METHYLESTERASE 2 (PME2)*, *GALACTURONOSYLTRANSFERASE 5 (GAUT5)*,* GALACTURONOSYLTRANSFERASE-LIKE 6 (GATL6)*, *GATL9*, *GATL10*, *XYLOGLUCAN ENDOTRANSGLUCOSYLASE/HYDROLASE 9 (XTH9)*, *XTH16*,* ARABINOGALACTAN PROTEIN 10 (AGP10)*, *AGP15*, *AGP16*, *AGP17*; and *TUBULIN BETA 7 (TUB7)*. We identified three lncRNAs upregulated during late shade treatment (45–90 min) in hypocotyl in this module: 1) NAT lncRNA *AT3G03265*, which has PIF4, PIF5 and PIF7 binding sites in its upstream region and is co-expressed with its overlapping gene the water channel *AQUAPORIN 1 (AQP1)* and which was also upregulated in cotyledons at 90–180 min. 2) NAT *AT5G09855*, which has upstream binding sites for PIF4 and PIF7, is upregulated at 45 vs. 90 min of shade treatment and overlaps the transcription factor *MYB DOMAIN PROTEIN 44 (MYB44)*, which is also upregulated in hypocotyl at this contrast. Furthermore, we identified evidence of MYB44 binding in the gene body of NAT *AT5G09855*, suggesting a possible regulatory feedback loop. 3) NAT *AtRegRNA.3868*, which overlaps the 3’ end of the putative actin cross-linking protein gene *AT1G27100*, was also expressed in the hypocotyl. PIF5 binds throughout the whole gene body of NAT *AtRegRNA.3868*. NAT gene *AT1TU033810* was previously identified in this region [27].

The genes in module P are drastically repressed in low R/FR in hypocotyls and enriched in functions related to the response to light stimulus, radiation and abiotic stimulus. This module includes the NAT lncRNA *AtRegRNA.18742*, which, like other genes in this module, is strongly downregulated at the latest treatment points (45–90 and 90–180 min) in the hypocotyl. *AtRegRNA.18742* is bound across its entirety by PIF3, and overlaps the 3’ end of the gene *AT3G05345*, which encodes a Chaperone DNA-j, and is similarly downregulated in 90 vs. 180 contrast and part of module P. A NAT transcript (*AT3TU005540*) in the same region was previously identified by [27]. This study also reported a NAT (*AT3TU090680*), overlapping the 5’ end of *ARATH; BSAS3;1* (*AT3G61440*, involved in the Cyanoamino acid metabolism; ath00460; module T). We also find a NAT lncRNA in that region (*AtRegRNA.26358*) whose upstream region is bound by PIF3, PIF4 and PIF5. This lncRNA also belongs to module P and, along with its antisense gene *ARATH; BSAS3;1*, is downregulated in hypocotyls at the latest timepoints of low R/FR exposure. *AtRegRNA.26,358* is co-expressed with genes such as *GH3.9*, *CWLP* and *XTH28*.

Module Q genes are drastically upregulated in hypocotyls in low R/FR and downregulated in cotyledons. This module contains the lncRNA *DANA1* (*AT4G06195*), which has upstream binding sites for PIF3 and PIF4 and increases its expression at the 45–90 min contrast in hypocotyls. The transcription factor *NF-YB3* (module A; downregulated at 90 vs. 180 min) and the auxin regulatory protein *IAA14* (module F, induced at 15 vs. 45 min and 45 vs. 90 min) change their expression in response to shade conditions and are both located within 10 kb of *DANA1*. Numerous genes related to cell wall remodeling and growth are coexpressed with *DANA1: XTH8*, *XTH22*, *XTH23*, *XTH33*, *GATL8*, *SAUR79*, *GAUT4*, *GAUT10*, *AGP2*, *EXPANSIN-LIKE 1 (EXPL1)*, *EXPL2*,* CSLC6*, *CSLC12*, *GLYCEROPHOSPHODIESTERASE-LIKE 2 (GPDL2)*, *AGP1,* and the brassinosteroid regulated kinase *THESEUS1 (THE1)*, which is required for cell elongation, suggesting that *DANA1* is involved in morphogenesis.

Module Q also contains the NAT *AtRegRNA.5500*, which has a PIF3 binding site in its upstream regulatory region, is upregulated in hypocotyl at 90 vs. 180 min, and overlaps at its 3’ end with the *cis* gene *SKU5-SIMILAR 6 (SKS6)* which is also upregulated at 90 vs. 180 min. A NAT gene, *AT1TU056750*, spanning the *SKS6* gene was identified previously [27]. This module also includes genes such as *CLIP ASSOCIATED PROTEIN (CLASP)* (involved in cell division and cell expansion), *GAUT10*, *GL2-EXPRESSION MODULATOR (GEM)* (involved in the spatial control of cell division), *CELLULOSE-SYNTHASE LIKE C6 (CSLC6)*, *FLOWER-SPECIFIC*,* PHYTOCHROME-ASSOCIATED PROTEIN PHOSPHATASE 3 (FYPP3)*, the callose synthase *GLUCAN SYNTHASE-LIKE 5 (GSL5)* and *REGULATORY PARTICLE NON-ATPASE SUBUNIT 5B (RPN5B)*, which encodes one isoform for the 26 S proteasome subunit RPN5B.

Genes in module AE first increase and later decrease in low R/FR in both cotyledons and hypocotyls. This module includes a NAT (*AtRegRNA.34273*) upregulated in hypocotyl (90–180 min), whose gene body is bound by PIF3 and PIF4. This NAT overlaps the 3’ end of *BES1-INTERACTING MYC-LIKE1 (BIM1)* (*AT5G08130*). *BIM1* is involved in brassinosteroid signaling and has been reported to positively regulate shade avoidance in *A. thaliana.* Interestingly, *BIM1* is also upregulated in the same contrast as its NAT but belongs to module R. Two NATs were previously found to arise from the same region: *NAT-lncRNA_1811* and *AT5TU009100* [27, 33]. We also found induced lincRNA *AT3G05945* in the 45 vs. 90 min contrast, which has PIF1, PIF3, PIF4, and PIF5 DNA binding motifs across the gene. This gene shows decreased expression in *OXIDATIVE STRESS 2* mutants in normal conditions, independent of salt stress [45].

**Functional evaluation of the effect of T-DNA insertions in or near lncRNA genes differentially expressed during the shade avoidance response**.

To further explore roles for lncRNAs in the shade avoidance response, we selected three lncRNA genes as candidates for functional analysis. *AT1G04877*, *AtRegRNA.1474.1* and *AtRegRNA.8746* were found in modules F and B with GO terms related to shade avoidance such as ‘growth’, ‘response to hormone’, ‘cell wall organization or biogenesis’, ‘developmental process’, and had T-DNA insertions listed in the Arabidopsis T-DNA insertion database (http://signal.salk.edu/cgi-bin/tdnaexpress*).AtRegRNA.1474.1* and *AtRegRNA.8746* were previously unannotated, while *AT1G04877* was annotated as a lncRNA in the Araport11 database (Table 1). lncRNA *AT1G04877* (upregulated at the 15–45 min contrast) is part of module F, while *AtRegRNA.1474.1* and *AtRegRNA.8746* (both downregulated at 90–180 min) are part of module B. All three of these lncRNAs are described in more detail in the previous section.

**Table 1:** Candidate lncRNAs for function in the shade avoidance response

ID	chr	start	end	strand	exons	module	Biological process related to shade (GO terms)	biotype	T-DNA	length
*AT1G04877*	chr1	3,960,943	3,961,245	+	1	F	‘growth’; ‘response to hormone’; ‘signaling’; ‘cell wall organization or biogenesis’	Intergenic	SALK_140097	302
*AtRegRNA.1474*	chr1	3,545,081	3,545,447	+	1	B	‘anatomical structure development’; ‘developmental process’; ‘response to hormone’	Intergenic	SAIL_69_F12C1	366
*AtRegRNA.8746*	chr1	24,911,749	24,913,729	-	3	B	‘anatomical structure development’; ‘developmental process’; ‘response to hormone’	Intergenic	SALK_127009	1980

Wild-type seed and seed homozygous for T-DNA insertions in these three lncRNA genes were grown on plates in a growth chamber in standard light conditions for 4 days and then transferred to either high or low R/FR conditions (Fig. S6 to see light spectrum) for 3 more days (see Materials and Methods). The lengths of hypocotyls and petioles of wt seedlings and T-DNA insertion mutants were then determined and compared between low and high R/FR conditions (see Materials and Methods for details). Seedlings homozygous for the SALK_140097 insertion in *AT1G04877* did not show a differential growth phenotype in high vs. low R/FR (Fig. S5). The absence of phenotype observed in this T-DNA line might be due to a lack of effect of the T-DNA insertion on the function of lncRNA *AT1G04877* or to a lack of function of this lncRNA in the shade avoidance response.

**Fig. 7:**
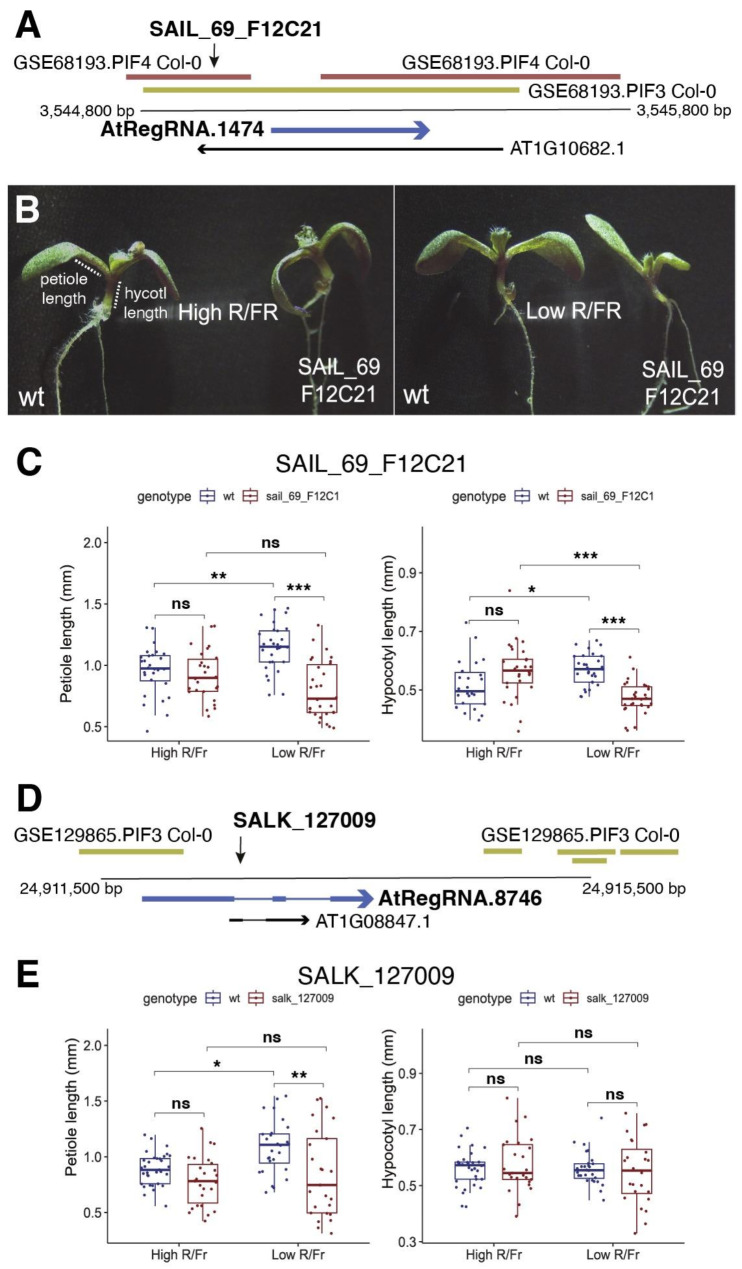
Characterization of lncRNAs *AtRegRNA.1474* and *AtRegRNA.8746* genomic regions, T-DNA insertions and their effect on the shade response in Arabidopsis seedlings. (**A**) Genomic region of *AtRegRNA.1474* (horizontal blue arrow), between 3,544,800 and 3,545,800 bp on chromosome 1. Vertical arrow points to the position of the SAIL_69_F12C1 T-DNA insertion. Green and red represent the ReMap track experiments for physical binding of PIF transcription factors to the putative lncRNA promoter. (**B**) Phenotypes of the wt and SAIL_69_F12C1 seedlings after 4 days of growth in standard conditions and then 3 days under high R/FR and low R/FR. (**C**) Measurements of wt and homozygous SAIL_69_F12C1 lines in petioles and hypocotyls of *A. thaliana* seedlings after 4 days of growth in standard conditions and then 3 days under high R/FR and low R/FR. (**D**) Genomic region of *AtRegRNA.8746* (horizontal blue arrow), between 24,911,500 and 24,915,500 bp on chromosome 1. Vertical arrow points to the position of the SALK_127009 insertion. Green and red represent the ReMap track experiments for physical binding of PIF transcription factors to the putative lncRNA promoter. (**E**) Measurements of petioles and hypocotyls of wt and homozygous SALK_127009 *A. thaliana* seedlings after 4 days of growth in standard conditions and then 3 days under high R/FR and low R/FR. An ANOVA test of data in (**C**) and (**E**) showed that there were significant differences between genotypes and between genotype: treatment interactions. p values for results of Tukey tests comparing any two given genotypes and treatments are shown in the panels: * *p* < 0.05; **p < 0.01; ***p < 0.001. *n* ~ 25 seedlings measured for each genotype and treatment

For *AtRegRNA.1474.1*, seedlings homozygous for the T-DNA insertion SAIL_69_F12C1 showed reduced petiole and hypocotyl length under low R/FR light compared to wt seedlings (Fig. 7A-C). The SAIL_69_F12C21 T-DNA is inserted just upstream of the *AtRegRNA.1474.1* transcript and in the exon of gene *AT1G10682*, which is annotated as a small ORF of unknown function but might also be a lncRNA. However, the transcript level of *AT1G10682* does not change between high and low R/FR conditions, while the transcript for *AtRegRNA.1474.1* is downregulated at the 90–180 min contrast (Supplemental Table 4), suggesting that the phenotype observed in SAIL_69_F12C2*1* is due to the alteration of function of *AtRegRNA.1474.1* and not *AT1G10682*. For the *AtRegRNA.8746*, we identified the SALK_127009 T-DNA insertion in the second intron of *AtRegRNA.8746*. Compared to the wild type, seedlings homozygous for SALK_127009 showed a reduction in petiole length in low vs. high R/FR light conditions (Fig. 7D and E). SALK_127009 also interrupts lncRNA *AT1G08847*, but this lncRNA is not differentially expressed between low and high R/FR conditions, while *AtRegRNA.8746* is differentially expressed at the 90 vs. 180-minute contrast, suggesting that the phenotypes observed in SALK_127009 are due to alteration of function of *AtRegRNA.8746* and not *AT1G08847*.

## Discussion

Here, we identified lncRNAs that likely contribute to the shade avoidance response in Arabidopsis. To accomplish this, we screened for lncRNAs with altered expression in cotyledons and hypocotyls of *A. thaliana* seedlings grown in high R/FR light and then switched to low R/FR light at 4 exposure times [31]. Most lncRNAs we identified ranged in length from 200 to 1000 nt, and were also observed in rice and *Brassica* species [46, 47]; most transcripts were mono-exonic like in rice [48]. This indicates that the transcripts identified as lncRNAs could indeed be *bona fide* non-coding genes. The larger number of differentially expressed lncRNAs in hypocotyls compared to cotyledons might be correlated with the dramatic growth differential in hypocotyls compared to cotyledons in shade conditions, while the fact that most lncRNAs we found were differentially expressed at mid or late timepoints suggests that lncRNAs may primarily play a role in differential growth as opposed to light perception or signaling.

A recent study by Li et al. (2021) also identified lncRNAs involved in the shade response, but in *Dendrobium officinale* [28]. Specifically, GO and KEGG analysis highlighted that differentially expressed lncRNAs in *D. officinale* were involved in pathways similar to those identified in our analysis (for example, ‘hormone signal transduction’), but not others such as ‘red and far-red light signaling pathway’ and other GO categories directly related to differential growth. Thus, our work expands on the study of Li et al. (2021) by identifying gene expression modules that are likely to be directly involved in the perception of shade and differential growth to shade. Furthermore, our study was performed in the highly tractable genetic model Arabidopsis, facilitating the functional characterization of lncRNAs in the shade avoidance response.

Most DE lncRNAs we found were in module E, containing GO functions related to shade avoidance such as ‘response to stimulus’, ‘response to organic substance’ and ‘response to chemical’. Previous work on the mechanisms of lncRNA function can illuminate mechanisms of how the lncRNAs we identified could act in a photomorphogenic context. *HID1* acts as a transcriptional repressor of *PIF3* [49], while under blue light conditions, *BLIL1* functions as a competing endogenous RNA of miR167, resulting in an increase in hypocotyl length in the *blil1* mutant [18]. The lncRNA *APOLO* that functions in shade responses was first described as a *cis* regulator of the *PINOID* gene [50] and a *trans* activator of auxin response and biosynthesis genes by forming R-loops within DNA [51, 52]. More recently, *APOLO* transcript levels were shown to be modulated by low FR light treatment to promote leaf hyponasty, a feature of the SAS response [53]. Finally, lncRNAs can be co-regulated with PIF transcription factors; for example, the lncRNA *PUAR7* physically associates with PIF7 to repress its *cis* gene *PHYA* [30].

Along these lines, some lncRNAs identified in this work were co-expressed with coding genes previously identified as PIF-dependent shade response genes. One example is the lincRNA *AT1G04877*, which is highly expressed in the hypocotyl [54], contains a PIF3 DNA binding motif in its promoter, and is co-expressed with the transcription factor gene *BHLH134* and the auxin-related genes *IAA4*, *SAUR28*, and *ARGOS* [55]. Another example is the sense-exonic lncRNA *AtRegRNA.29,116*, which was co-expressed with *SAUR68* and also has a PIF3 binding site in its regulatory region. Both *AT1G04877* and *AtRegRNA.29,116* are in the F module, which contains GO terms such as response to hormone, growth, and signaling. While our analysis of the SALK_140047 T-DNA insertion in *AT1G04877* didn’t find a phenotypic effect, a function for *AtRegRNA.29,116* in the SAS remains to be tested. In the case of *AT1G04877*, it is possible that the T-DNA insertion we examined does not affect lncRNA stability. In the future, both of these lncRNAs should be tested for function using knock-down lines.

Regarding the lncRNAs *AtRegRNA.1474.1* and *AtRegRNA.8746*, we found that T-DNA insertions in or near these lncRNAs did condition phenotypes in the SAS response. In the case of *AtRegRNA.1474.1*, the T-DNA insertion SAIL_69_F12C21 caused an opposite phenotype to the shade response, showing a reduction in the petiole and hypocotyl length compared to wild-type seedlings. SAIL_69_F12C21 falls in the putative promoter region of *AtRegRNA.1474.1*, interrupting DNA binding sites for the transcriptional regulators PIF3 and PIF4. Thus, the T-DNA insertion in this region probably affects the binding of PIF3 and PIF4 to *PIF3* and/or *PIF4* enhancer sites, decreasing transcription of *AtRegRNA.1474.1.* This case would be similar to *SEAIRa*, where a phenotype was observed by deleting part of the promoter region using the CRISPR/Cas9 system [56]. However, more studies are needed to unequivocally link the phenotype produced by the SAIL_69_F12C21 insertion with lncRNA *AtRegRNA.1474.1*, because SAIL_69_F12C21 also falls in the 3’ end of *SHORT OPEN READING FRAME 1* (*AT1G10682*), a translated open reading frame of unknown function.

In the case of *AtRegRNA.8746*, we found that the T-DNA insertion SALK_127009, which is located in the first intron of this lncRNA, conditions a phenotype of shorter petioles compared to wild type under low R/FR conditions. Though *AtRegRNA.8746* contains PIF3 binding sites both in the first exon and downstream of the gene, the location of SALK_127009 in the first intron suggests that the effect of this T-DNA on *AtRegRNA.8746* is most likely to affect the stability or splicing of the mRNA. Though of course, it is possible that SALK_127009 causes an early transcriptional termination of *AtRegRNA.8746* due to the T-DNA insertion in the intron, which is likely to be at least 10 kb in length. *AtRegRNA.8746* overlaps *AT1G08847*, a predicted lncRNA of unknown function, and the SALK_127009 T-DNA is inserted in the first exon of *AT1G08847*. As noted above in the results, the expression of *AT1G08847* does not change between low and high R/FR conditions, while the expression of *AtRegRNA.8746* does change, making it more likely that the lack of *AtRegRNA.8746* function is responsible for the phenotype observed in SALK_127009. Nonetheless, to unequivocally demonstrate that the lack of *AtRegRNA.8746* function is responsible for decreased petiole growth in low R/FR conditions, it will be necessary to differentiate between these two lncRNA genes in the SAS response, perhaps by adding back the function of only AT1G08847 to the SALK_127009 line, to determine if this rescues the SAS phenotype.

## Conclusions

In conclusion, we have identified lncRNAs participating in shade avoidance induced by far-red light (low R/FR). Of all the differentially expressed lncRNAs, we picked 3 candidates for function in shade avoidance, and we identified phenotypes in response to low R/FR light conditions for 2 out of 3 T-DNA insertions located in or near these lncRNAs. The phenotypes conditioned by these two T-DNA insertions suggest that the corresponding lncRNAs function in the shade avoidance response and validate our computational approach to identifying lncRNAs potentially involved in shade avoidance. Thus, *AtRegRNA.1474.1* and *AtRegRNA.8746* can be added to the list of lncRNAs with potential roles in growth and development. Beyond the probable involvement of these two lncRNAs in the SAS, this result further adds to the mounting evidence that, of the thousands of lncRNAs in the Arabidopsis genome, many of them do indeed have functions and are not just transcriptional side products [57].

In the future, additional assays will be required to unequivocally establish a role for these novel lncRNAs in a specific mechanism underlying the SAS phenomenon. For example, part of the shade avoidance response is to increase auxin production to promote hypocotyl elongation, and two lncRNAs have previously been reported to regulate auxin perception or response. *AUXIN REGULATED PROMOTER LOOP (APOLO)* is a lncRNA induced by auxin, which causes demethylation in the neighboring *PINOID (PID)* gene, promoting PID expression and differential growth based on auxin perception [50]. A second work reported that *APOLO* recognizes spatially distant auxin response genes by forming R-loops [51]. We hypothesize that the lncRNAs identified in our study may regulate auxin response genes, or other genes required for differential growth during the shade avoidance response.

### Electronic supplementary material

Below is the link to the electronic supplementary material.

Supplementary Material 1

## Data Availability

The raw RNA-seq data used for the analyses in this study were produced by Kohnen et al., (2016) and are available at the National Center for Biotechnology Information Gene Expression Omnibus (GEO) as GSE81202.
