# Supplementary material for: lncRNAs involved in the Shade Avoidance Syndrome (SAS) in Arabidopsis thaliana
Source: BMC Genomics. 2024 Aug 26;25:802. doi: 10.1186/s12864-024-10718-z (PMC11346216; doi:10.1186/s12864-024-10718-z)
Supplement: Supplementary file 1 — Supplementary Material 1 [file 12864_2024_10718_MOESM1_ESM.pdf]

Supplementary Material

lncRNAs involved in the Shade Avoidance Syndrome (SAS) in *Arabidopsis thaliana*

Irving García-López[1], Aarón I. Vélez-Ramírez[2, 3], C. Stewart Gillmor[1]* and Selene L. Fernandez-Valverde[4,5,6]*

1 Unidad de Genómica Avanzada, Cinvestav, Irapuato, Guanajuato, 36824, México

2 Laboratorio de Ciencias Agrogenómicas, Escuela Nacional de Estudios Superiores Unidad León, Universidad Nacional Autónoma de México, León, Guanajuato, 37684, México

3 Laboratorio Nacional PlanTECC, Escuela Nacional de Estudios Superiores Unidad León, Universidad Nacional Autónoma de México, León, Guanajuato, 37684, México

4 School of Biotechnology and Biomolecular Sciences, The University of New South Wales, 2052, Sydney, NSW, Australia

5 UNSW RNA Institute, The University of New South Wales, 2052, Sydney, NSW, Australia

6 Evolution & Ecology Research Centre, The University of New South Wales, 2052, Sydney, NSW, Australia

* Correspondence: stewart.gillmor@cinvestav.mx, s.fernandez_valverde@unsw.edu.au

Supplemental Figures

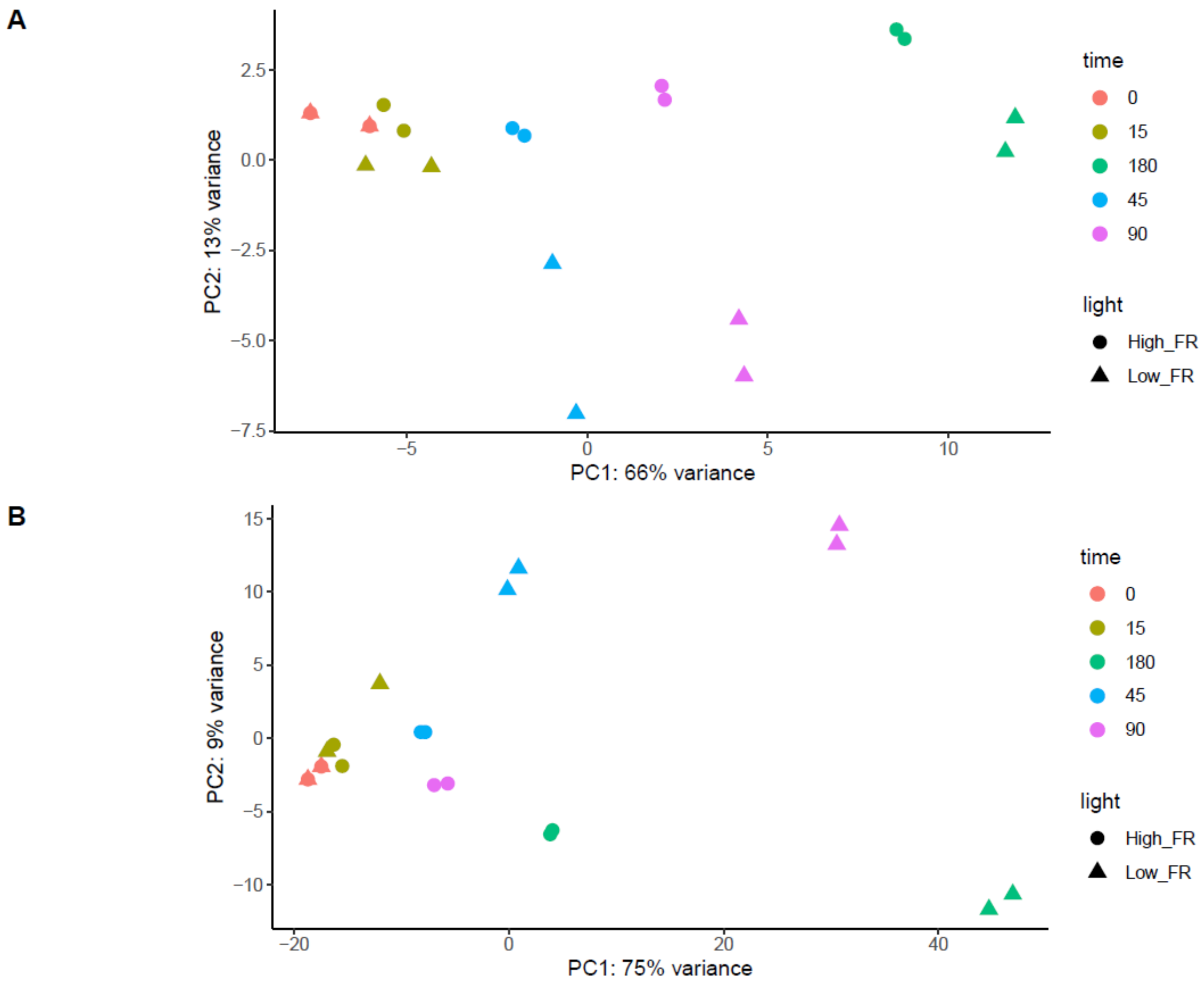

Figure S1. PCA of **A)** Cotyledon and **B)** Hypocotyl libraries by eliminating genes with low counts. Principal Component 1 (PC1) and PC2 are represented.

Tissue	contrasts	Biotype	log2fc	Genes
Cotyledon	15vs45	NAT	up	1
Cotyledon	15vs45	Intergenic	up	1
Cotyledon	45vs90	NAT	down	3
Cotyledon	45vs90	NAT	up	1
Cotyledon	45vs90	Intergenic	down	2
Cotyledon	90vs180	NAT	up	4
Cotyledon	90vs180	Intergenic	up	1
Cotyledon	90vs180	Sense-exonic	down	1
Cotyledon	90vs180	Sense-exonic	up	1
Hypocotyl	15vs45	NAT	down	3
Hypocotyl	15vs45	NAT	up	2
Hypocotyl	15vs45	Intergenic	down	1
Hypocotyl	15vs45	Intergenic	up	1
Hypocotyl	45vs90	NAT	down	19
Hypocotyl	45vs90	NAT	up	6
Hypocotyl	45vs90	Intergenic	down	13
Hypocotyl	45vs90	Intergenic	up	6
Hypocotyl	45vs90	Sense-exonic	down	2
Hypocotyl	45vs90	Sense-exonic	up	2
Hypocotyl	90vs180	NAT	down	49
Hypocotyl	90vs180	NAT	up	17
Hypocotyl	90vs180	Intergenic	down	21
Hypocotyl	90vs180	Intergenic	up	6
Hypocotyl	90vs180	Intronic	down	1
Hypocotyl	90vs180	Sense-exonic	down	6
Hypocotyl	90vs180	Sense-exonic	up	5

Table S1. LncRNA expression classified according to genomic context. Number of f lncRNAs genes DE induced and repressed (*padj* < 0.01) in each time contrast (minutes) during shade treatment in cotyledon and hypocotyl tissue.

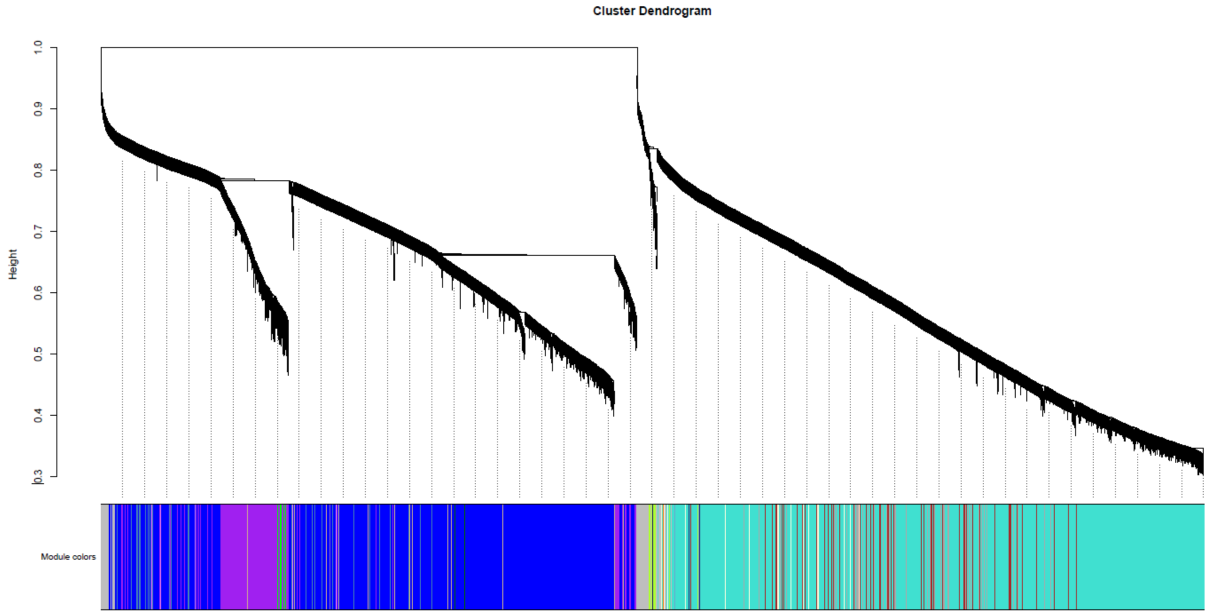

Figure S2. WGCNA analysis in automatic mode. Gene grouping dendrogram (coding and lncRNAs), with gene dissimilarity based on topological overlap with the color module to which each gene was assigned. Each color is clustered as a module. Key colors and how many genes does it contain (A = turquoise 3325; B = blue 2873; C = brown 1303; D = yellow 1229; E = green 1180; F = red 1068; G = black 769, H = pink 762; I = magenta 749; J = purple 724; K = greenyellow 717; L = tan 704; M = salmon 699; N = cyan 688; O = midnightblue 604; P = lightcyan 578; Q = grey60 415; R = lightgreen 365; S = lightyellow 357; T = royalblue 289; U = darkred 240; V = darkgreen 275; W = darkturquoise 273; X = darkgrey 240, Y = orange 231, Z = darkorange 224; AA = white 220; AB = skyblue 185; AC = saddlebrown 180; AD = steelblue 178; AE = paleturquoise 147; AF = violet 110; AG = darkolivegreen 59; AH = darkmagenta 43; AI = sienna3 36; AJ = yellowgreen 36; AK = skyblue3 33; AL = plum1 24; AM = orangered4 22).

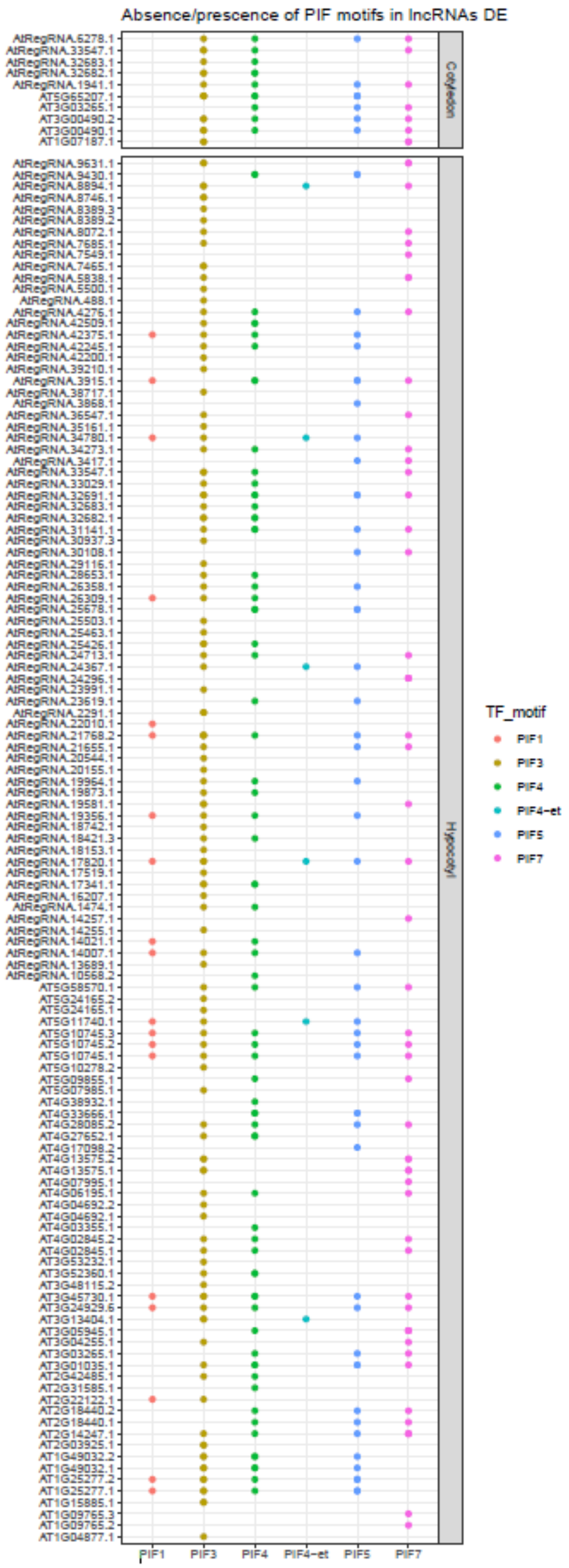

Figure S3 Absence/Presence of PIFs motifs in the upstream region of DE lncRNAs. Using a 2000 region as a promoter for each lncRNA. Circles represent a hit of partial or complete DNA physical binding to DNA by PIFs (PIF1, PIF3, PIF4, PIF5 and PIF7; PIF4-et etiolated experiment).

Figure S4 available on FigShare due to space constraints - 10.6084/m9.figshare.25745190

Figure S4 Representative expression of co-expression modules. Representative expression (eigengene, y-axis) for co-expression modules of over 20 genes (top labels), across the time course (x-axis). Each replicate is shown in a different colour. Cotyledons are shown in the first two rows and Hypocotyls in the lower two rows, alternating between High R/FR (rows 1,3) and Low R/FR (rows 2,4). Low R/FR rows lack data on time 0; this was only sequenced once in the original study for each tissue.

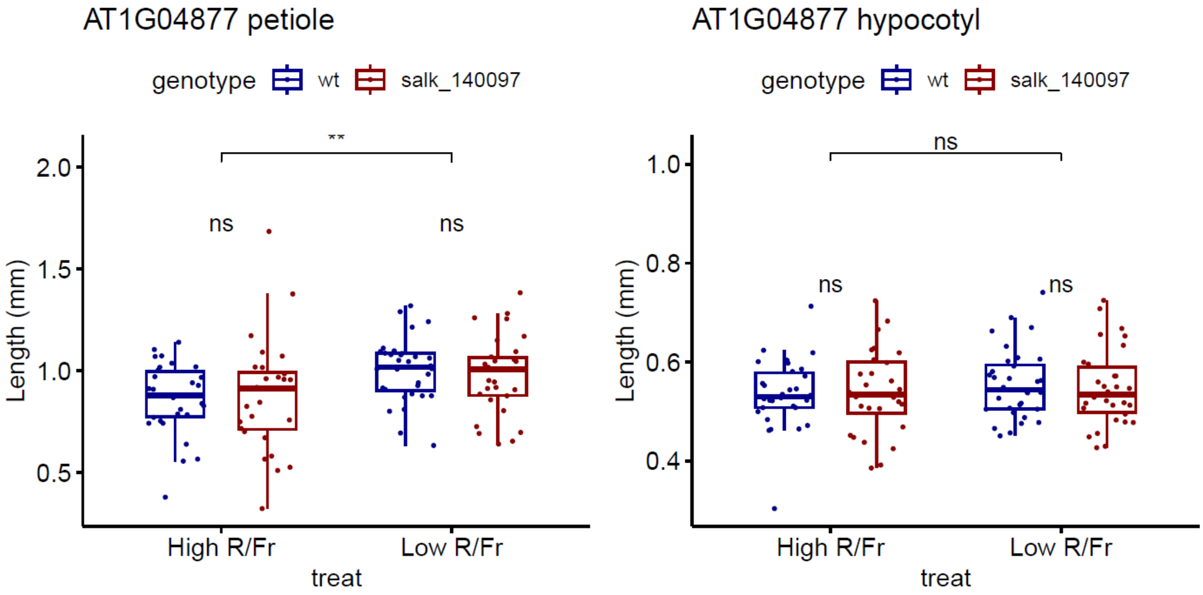

Figure S5. Measurements of the AT1G04877 lncRNA in petioles and hypocotyls of *A. thaliana* seedlings under High and Low R/FR light. Four-day Arabidopsis seedlings exposed at 3 days in High and Low R/FR. WT and mutant seedlings (salk_140097) were analyzed. Two-way anova test, n = ~25, * $p < 0.05$.

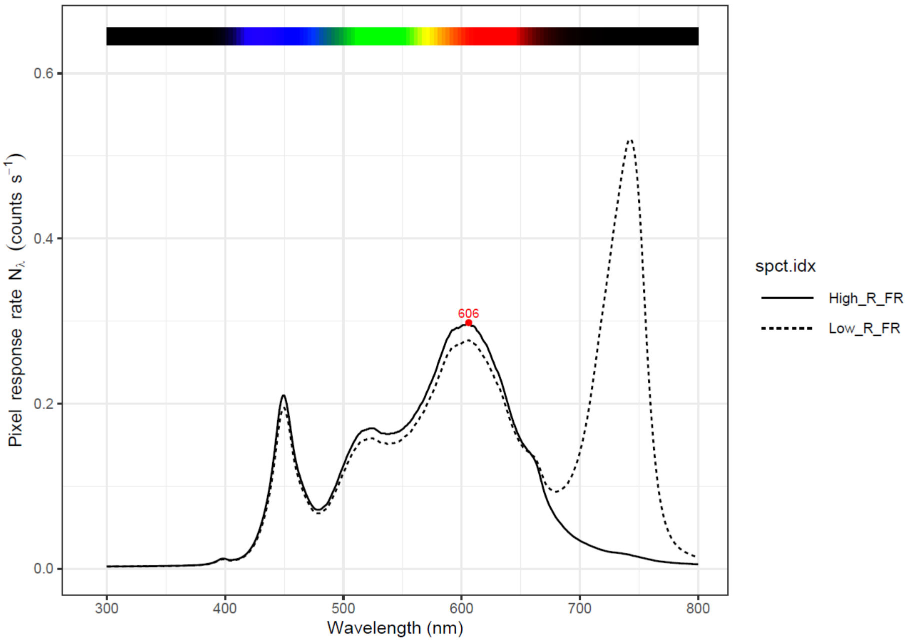

Figure S6. Spectral plot of high and low R/FR chambers. The continuous line represents the full spectrum of the lamp without Far Red LEDs and the dotted line represents the lamp with Far Red LEDs. The spectrum for each lamp was adjusted to a photosynthetically active photon flux of 150 µmol m^{-2} s^{-1} . For this plot the "ggspectra" library was used [1].

References: 1. Aphalo PJ. The r4photobiology suite: spectral irradiance. UV4PB. 2015;2015:21–9.
